# DNA Fingerprinting of Mycobacterium tuberculosis Isolates from Epidemiologically Linked Case Pairs

**DOI:** 10.3201/eid0811.020420

**Published:** 2002-11

**Authors:** Diane E. Bennett, Ida M. Onorato, Barbara A. Ellis, Jack T. Crawford, Barbara Schable, Robert Byers, J. Steve Kammerer, Christopher R. Braden

**Affiliations:** * Centers for Disease Control and Prevention, Atlanta, Georgia, USA

**Keywords:** *Mycobacterium tuberculosis*, DNA fingerprinting, molecular epidemiology, contact investigation

## Abstract

DNA fingerprinting was used to evaluate epidemiologically linked case pairs found during routine tuberculosis (TB) contact investigations in seven sentinel sites from 1996 to 2000. Transmission was confirmed when the DNA fingerprints of source and secondary cases matched. Of 538 case pairs identified, 156 (29%) did not have matching fingerprints. Case pairs from the same household were no more likely to have confirmed transmission than those linked elsewhere. Case pairs with unconfirmed transmission were more likely to include a smear-negative source case (odds ratio [OR] 2.0) or a foreign-born secondary case (OR 3.4) and less likely to include a secondary case <15 years old (OR 0.3). Our study suggests that contact investigations should focus not only on the household but also on all settings frequented by an index case. Foreign-born persons with TB may have been infected previously in high-prevalence countries; screening and preventive measures recommended by the Institute of Medicine could prevent TB reactivation in these cases.

Investigating persons who have had close contact with tuberculosis (TB) cases is an essential element of public health programs to control and eliminate TB (1,2). These contact investigations are done primarily to discover persons who may require treatment for latent TB infection and also to find and treat additional persons with TB. While not usually highly contagious, TB is generally transmitted to persons who have shared indoor air space frequently or for a long period of time with a person who is infectious (3). Factors that may influence transmission include prolonged hours of contact during the infectious period, close proximity to the person with TB, and lack of ventilation and ultraviolet light in a shared environment. Generally, close contacts who live with a person identified with active TB or who habitually spend time indoors in close proximity to this person are investigated first. If no evidence of TB transmission is found in these close contacts, the investigation ceases. If transmission has occurred, the investigation may be extended. The “stone-in-the-pond” principle, a technique in which concentric circles of contact persons around the case are sequentially investigated, is practiced in many countries (4).

If one or more additional persons with TB are identified among the contacts of a person with TB, the person is labeled as the index case for the purpose of the investigation; those subsequently identified are classified as source cases, secondary cases, or unlinked cases. An active TB case found in a contact investigation may be classified as the source of infection to the index case, a secondary case infected by the index case, or a case who neither infected nor was infected by the index case (with a strain of TB unrelated to the strain of the index case). Information about the start and duration of symptoms for the index and the contact cases, and the start and duration of contact between them, facilitates categorization. Categorizing a contact with active TB as a source case, a secondary case, or an unlinked case, based on epidemiologic evidence, helps to direct further investigation. If the source case is known to have drug-resistant TB, establishing epidemiologic links may also aid in choosing an appropriate drug regimen for the contact before cultures and sensitivity test results are available (5).

The chief priority of TB-control programs is to identify and treat active cases of TB before transmission can occur (1,3). If an index case is identified and treated soon after symptoms begin, the time during which TB could be transmitted can be minimized, and active secondary cases are unlikely to be found in contact investigations. If contact investigations are carried out soon after the index case is identified, the time is minimized during which infected persons could progress to disease before receiving treatment for latent TB infection to prevent progression. In a low-prevalence country, a well-resourced and active TB-control program would not expect to find a high proportion of active TB cases among contacts in investigations.

Systematic evaluations of contact investigations are infrequent. In an Australian study, of 1,142 close contacts of 231 cases diagnosed in 1991, a mean of 4.9 contacts per case were identified, but only 3 (0.3%) of the contacts had active TB (6). However, the authors stated that the screening of these contacts was inadequate so TB may have been underdiagnosed. A 1996–1997 U.S. study of contact investigations of 1,080 pulmonary, smear-positive TB patients found a median of four close contacts per patient (7). Thirty-six percent of contacts were tuberculin skin-test positive, and 2% had active TB. A systematic review of health department records for all contacts of 349 patients with culture-positive TB in five study areas in 1996 revealed that 13% had not identified contacts (8). Although 3,824 contacts were identified, only 2,095 (55%) completed screening; of these, 1% had active TB.

DNA fingerprinting has been used to support contact investigations of large clusters of cases in institutional settings and to suggest possible connections among cases without obvious epidemiologic links. This technique is also used, though rarely, to evaluate epidemiologic links found in contact investigations. In San Francisco, culture-positive TB cases previously identified as contacts to active TB cases were evaluated along with their index cases (9); a median of four contacts was investigated for each of the 1,308 culture-positive index cases reported from 1991 to 1996. Of 11,211 contacts evaluated, 108 (1%) had active TB. Of 94 pairs of index and contact cases with active TB, 66 had positive cultures; of these, 54 had restriction fragment length polymorphism results for both strains. Transmission was confirmed (that is, the same strain was identified in both cases) in 38 (70%) of the 54 pairs.

Between 1996 and 2000 in the United States, the National Tuberculosis Genotyping and Surveillance Network collected information on contacts with culture-confirmed TB identified during the course of contact investigations and medical record reviews in seven sentinel areas (the states of Arkansas, Massachusetts, Maryland, Michigan, and New Jersey and selected sites in California and Texas). These data, combined with the results of DNA fingerprinting, were analyzed to evaluate the proportion of epidemiologically linked cases in which transmission was confirmed by matching fingerprints and to investigate the characteristics of case pairs with unconfirmed transmission (unmatched fingerprints).

## Methods

### Epidemiologic Information from Contact Investigations

Details regarding the study population are presented elsewhere (10,11). Information captured from routine contact investigations and medical record reviews (done before fingerprinting data were available) were entered into an Epi Info version 6.03 database (Centers for Disease Control and Prevention [CDC], Atlanta, GA) and sent quarterly to CDC. Participating sites entered information on any index cases reported as a definite TB case to the state TB surveillance system from January 1996 to December 2000 and on any contact to an index case reported as a definite TB case between January 1990 and December 2000.

 Because the study was directed at an evaluation of information acquired during routine public health procedures, information acquired in cluster investigations done because of DNA fingerprint clustering was not included. Information from epidemiologic cluster investigations conducted because more than one case of TB was noted in a congregate setting, before fingerprinting had been performed, was included.

Participating sites recorded the nature of the relationship between the index case and each contact, the setting in which the two persons interacted, and the direction of transmission (i.e., whether the contact was identified as the source case in relation to the index case, a secondary case in relation to the index case, or whether the direction of transmission remained unidentified). In our analysis, we included only case pairs in which the direction of transmission was specified.

### Demographic and Clinical Information

Demographic and clinical information on source and secondary cases was noted by matching state case numbers to the national TB surveillance system database at CDC. Data reported included gender, race/ethnicity, age at diagnosis, country of origin, previous episodes of TB, sputum smear status at diagnosis, chest x-ray results, drug susceptibility results, HIV status, and occupation.

### Laboratory Methods

 In this study, a source case was defined as a person with active TB identified in a routine contact investigation as the probable source of transmission to another person with active TB. A secondary case was a person with active TB identified in a routine contact investigation as having acquired TB from one source case. An epidemiologically linked case pair consisted of two persons identified respectively as linked source and secondary case in the course of a contact investigation. A secondary case could not be linked to more than one source case. However, one or more secondary cases could be linked to a single source case.

Transmission was considered confirmed if matching DNA fingerprint patterns were found in both isolates from a case pair. We attempted DNA fingerprinting for all available isolates from participating sites during the period of the study. The methods we used, including a definition for matching fingerprint patterns, are described in a related article (10).

### Statistical Methods

Univariate associations between demographic, setting, and clinical variables, and the dependent variable (TB transmission unconfirmed by DNA fingerprinting) were examined by using the Cochran-Mantel-Haenszel test for unequal odds using SAS version 8.0 (SAS Institute, Inc., Cary, NC). Associations were also examined by multiple logistic regression analysis in SPSS version 6.0 (SPSS, Inc., Chicago, IL). The correlation matrix produced was examined for potential colinearity between variables. Goodness-of-fit analysis was performed by using the SPSS Hosmer-Lemeshow goodness-of-fit test (12). To choose the best-fitting model, we used the -2 log-likelihood ratios test to compare models. A p value <0.05 was considered statistically significant.

## Results

### Source and Secondary Cases in Epidemiologically Linked Case Pairs

Contact investigations in the sentinel sites identified 538 epidemiologically linked case pairs in which a direction of transmission was specified, both cases were culture positive, and fingerprints were available for both isolates (Table 1). These pairs included 397 source cases, of which 324 (82%) were linked to only 1 secondary case. Of the remaining 73 source cases, 48 were linked to 2 secondary cases; the rest were linked to between 3 and 11 secondary cases.

**Table 1:** Characteristics of source and secondary cases in epidemiologically defined tuberculosis case pairs from seven states, 1996–2000

Characteristic	Tuberculosis case pairs (%) (n=538)	
	Source cases (%) (n=397)	Secondary cases (%) (n=538)
Gender		
Men	62	60
Women	38	40
Race/ethnicity		
White	16	16
Black or African-American	51	56
Hispanic	17	15
Asian or Pacific Islander	16	13
Age		
<15 yrs	1	14
15–44 yrs	61	54
44–65 yrs	28	26
>65 yrs	9	7
History of previous tuberculosis	7	3
Foreign-born	35	25
Pulmonary disease	95	91
Negative sputum smear	25	47
Cavitary disease	47	22
Homeless	9	7
Alcoholic	27	22
Injection drug user	6	5
HIV seropositive	11	9

### Factors Associated with Transmission Unconfirmed by DNA Fingerprinting

Transmission was not confirmed (source and secondary cases had different fingerprints) in 156 (29%) of the 538 epidemiologically linked case pairs. This proportion was unchanged for the 260 case pairs in which both persons lived in the same household; transmission was unconfirmed in 80 (31%) of these pairs.

The results of univariate analysis comparing case pairs with unconfirmed transmission to case pairs with confirmed transmission are shown in Table 2. Case pairs with unconfirmed transmission were as likely to be from the same household as those with confirmed transmission, but were more likely to be identified through a shared workplace (odds ratio [OR] 2.3). In univariate analysis, case pairs with unconfirmed transmission were more likely to include a smear-negative source case (OR 2.4), a foreign-born secondary case (OR 4.7), and a case pair in which both persons were Asian or Pacific Islanders (OR 3.5) than case pairs with confirmed transmission. Case pairs with unconfirmed transmission were less likely to include a secondary case <15 yrs of age (OR 0.4) and a case pair in which both persons were black or African-American (OR 0.5) than pairs with confirmed transmission.

**Table 2:** Univariate analysis of factors associated with transmission unconfirmed by DNA fingerprinting in tuberculosis case pairs identified in contact investigations in seven sites, 1996–2000

Variable	Transmission unconfirmed (%) (n=156)	Transmission confirmed (%) (n=382)	Odds ratio (95% confidence intervals)	p value^a^
Relationship between persons in case pairs				
Shared household	80 (51)	180 (47)	1.2 (0.8 to 1.7)	NS
Friends/social	55 (35)	156 (41)	0.8 (0.5 to 1.2)	NS
Shared workplace	11 (7)	12 (3)	2.3 (1.1 to 5.4)	0.04
Other	10 (6)	34 (9)	1.0 (0.6 to 1.5)	NS
Characteristics of source cases				
Sputum smear-negative	56 (36)	73 (19)	2.4 (1.6 to 3.6)	<0.0001
Cavitary disease	64 (41)	197 (52)	0.7 (0.5 to 1.0)	NS
Characteristics of secondary cases				
Foreign-born	73 (47)	60 (16)	4.7 (3.1 to 7.2)	<0.0001
Previous tuberculosis	5 (3.0)	12 (3.0)	1.1 (0.4 to 3.7)	NS
<15 yrs	8 (5)	65 (17)	0.4 (0.2 to 0.8)	0.007
>65 yrs	16 (11)	21 (6)	1.8 (0.9 to 3.6)	NS
HIV positive	13 (8)	33 (9)	1.0 (0.5 to 1.9)	NS
Characteristics of case pairs				
Both white	14 (9)	42 (11)	0.5 (0.4 to 1.5)	NS
Both black	62 (40)	221 (58)	0.5 (0.3 to 0.7)	0.005
Both Hispanic	21 (14)	46 (12)	1.1 (0.7 to 2.0)	NS
Both Asian or Pacific Islander	35 (22)	29 (8)	3.5 (2.1 to 6.0)	<0.0001
Racial discrepancy	24 (15)	40 (11)	1.2 (0.9 to 1.4)	NS
Age difference >10 yrs	88 (56)	198 (52)	1.2 (0.8 to 1.7)	NS

^a^NS, not significant.

All levels of any factor substantially associated (positively or negatively) with the unconfirmed transmission in the univariate analysis were entered into a multiple logistic regression model. The HIV status of secondary cases and cavitary disease in source cases was also included because of potential biologic importance. No interactions were seen, and interaction terms were removed. In the full model without interaction terms, case pairs with unconfirmed transmission were significantly more likely to include a smear-negative source case (OR 2.0) or a foreign-born secondary case (OR 3.4), and significantly less likely to include a secondary case <15 yrs of age (OR 0.3) than case pairs with confirmed transmission. The association for workplace setting seen on univariate analysis was not significant in this model (OR 2.7, p=0.08). We did not find a high level of colinearity between pairs of variables. Confidence intervals and p values for factors significantly associated with unconfirmed transmission in this model are shown in Table 3.

**Table 3:** Logistic regression model^a^ showing factors associated with transmission unconfirmed by DNA fingerprinting in tuberculosis case pairs identified in contact investigations in seven sites, 1996–2000

Factor	Adjusted odds ratio (95% confidence intervals)	p value
Smear-negative source case	2.0 (1.2 to 3.1)	0.001
Foreign-born secondary case	3.4 (2.0 to 6.0)	<0.0001
Secondary case <15 yrs	0.3 (0.2 to 0.8)	0.01
Secondary case >65 yrs	1.7 (0.8 to 3.6)	0.2
Workplace setting	2.7 (0.9 to 5.7)	0.08

^a^The model also included in the multiple logistic regression analysis all levels of the following factors that were not significantly associated with unconfirmed transmission: cavitary disease in source case, race/ethnicity of case pair, and HIV status of secondary case. In the goodness-of-fit test, full model’s p value was 0.885; and the p value for a reduced model containing only the variables with significant associations was 0.725. Thus, both models fit adequately. For the full model, –2 x log likelihood was 564.044; for the reduced model, –2 x log likelihood was 571.469. The difference was distributed as a chi-square variable with 1 degree of freedom. The full model gave a better fit than the reduced model and was retained (p=0.0064, chi square >7.425).

The DNA fingerprints of 34 nonmatching case pairs differed by fewer than three bands. When we removed these case pairs from the analysis, we found the same variables were still substantially associated with unconfirmed transmission, although the ORs differed slightly (results not shown), so these pairs were retained.

## Discussion

 Our study found that 29% of TB case pairs identified in contact investigations in seven sites during the course of 5 years were not confirmed as linked by using DNA fingerprinting. We used a restricted definition for considering a case pair to be epidemiologically linked: direction of transmission had to be identified as a result of the epidemiologic investigation. Despite the differences in methodology between our research and a California study (9), which used a less restrictive definition (an epidemiologic case pair was an index case and contact case), both studies found a similar percentage of unconfirmed case pairs.

 The fact that case pairs in which transmission was unconfirmed were no less likely to be found in the same household than case pairs in which transmission was confirmed suggests that apparent household transmission should not be taken at face value. Marks et al., in a review of U.S. contact investigations (7), report that 33% of contact investigations focused only on the household of the index case. Veen, however, suggests that a modern day stone-in-the-pond approach should focus first on the places and groups frequented by the person classified as the index case, which may not only be the household (4). Onorato notes that as TB in the United States becomes less prevalent, forms and settings of transmission previously considered unusual will appear relatively more common (13). Many public health departments have designed contact investigation formats to focus on places frequented by persons newly diagnosed with TB and the persons with whom they have had contact during the infectious period, rather than focusing only on the household (S. Sharnprapai, pers. comm.). Our findings suggest that this strategy may be useful, even when an apparent source case has been located in the same household.

 Unconfirmed transmission between an epidemiologically linked case pair has two interpretations: either the secondary case actually has a reactivation of a latent, remotely acquired TB infection or the transmission was recent, but the source case has not been identified. The progression of a latent, remotely acquired infection to active TB implies that an opportunity to identify infection and apply appropriate preventive measures may have been missed. Failure to correctly identify the source of a recent transmission suggests that contact-tracing procedures may have been inadequate or that an unusual setting or mode of transmission may have been associated with the infection. The significant associations found in our study suggest that either explanation could apply to many of the unconfirmed transmissions we identified.

 Few secondary cases in the study reported previous active TB, and prevalence of previous TB was equal among the confirmed and unconfirmed transmission groups. However, previous TB infection could still be an important factor among some foreign-born persons classified as secondary cases in case pairs in which transmission was unconfirmed. Foreign-born persons from countries with high TB prevalence are likely to have had multiple opportunities for exposure to TB before arrival in the United States (3), diminishing the likelihood that any one identified exposure, such as the source case identified in the contact investigation, is the source of transmission. Screening new arrivals from countries with a high prevalence of TB and scheduling additional screening of persons from such countries were both given a high priority in the Institute of Medicine’s recent recommendations for eliminating TB in the United States (3). The Institute noted that such screening is currently inadequate and warrants expansion, as well as follow-up to ensure that foreign-born persons with positive tuberculin skin tests receive treatment. Much of the active TB among the 133 foreign-born persons classified as secondary cases in this study, as well as some of the TB among the 139 foreign-born persons classified as source cases, might have been prevented if such screening and follow-up were more widely implemented.

 Another major finding in this survey is the significant positive association between a smear-negative source case and unconfirmed transmission. This finding suggests that identifying a smear-negative source case for an index case should not preclude ongoing investigation of other possible sources.

On the other hand, the study does not suggest that transmission from smear-negative cases does not occur. Contact investigations of smear-negative cases generally have a low priority in most public health departments. Given that contact investigations were not likely to have been conducted for many smear-negative cases in the network, the fact that 19% of confirmed case pairs had a smear-negative source case suggests that transmission from smear-negative source cases is not negligible. This proportion is similar to that found in a DNA fingerprinting study of two types of epidemiologic clusters, those with smear-negative source cases and those with smear-positive index cases (14). Twenty percent of case pairs with confirmed transmission had a smear-negative index case.

 Eighty-nine percent of case pairs with a secondary case <15 years of age were confirmed by genotyping. The shorter incubation of TB in children than in adults and the more limited social circles of children increase the likelihood that children with TB were infected within the circle of present contacts located in a contact investigation. These same factors make TB in children preventable. If TB in the 55 adult source cases who transmitted the infection to these children had been diagnosed and treated promptly and contact tracing had resulted in timely and complete treatment for latent TB infection, latent TB infection in most of these children would have been prevented or would not have progressed to TB.

 We hypothesized that secondary cases >65 years of age might have had a greater risk for unconfirmed transmission, given longer lives which included the era of relatively high TB rates before the availability of TB drugs. However, case pairs with unconfirmed transmission were no more likely to include an elderly person as a secondary case than pairs with confirmed transmission. TB in an elderly person should not be assumed to be a reactivation, and contact investigations should attempt to identify a possible source case.

Nearly half the workplace case pairs did not have transmission confirmed by DNA fingerprinting, although the number of case pairs was small. When active TB is diagnosed in a person in a workplace, screening is often not confined to close contacts. Casual contacts may be screened because of anxiety on the part of management and staff. Including nonclose contacts in the screening increases the likelihood that if an additional case is found, transmission did not occur from the index case.

 Our hypothesis that secondary cases with HIV are more likely to be part of a confirmed case pair than other secondary cases, since their compromised immune status predisposes them to a quicker progression to active TB once infected, was not borne out in this study. Persons with HIV are also more likely to reactivate previous TB infections than persons not dually infected, which may explain the relatively equal proportions of confirmed and unconfirmed transmissions from the identified source among HIV-positive persons. HIV may also be underreported in the TB surveillance system, and the numbers reported may not represent the real numbers of persons with HIV.

 Although DNA fingerprinting provided helpful research information in our study, we think the tool should not be considered a routine contact investigation tool. Contact investigations should ideally be undertaken, and often will be completed, before the isolates from the index case are available. Since early identification of TB cases and prevention of further cases is the highest priority, DNA fingerprinting methods will not be relevant to most contact investigations in a TB control program. If TB cases are identified early and appropriate preventive measures taken for latent TB infection seen in contacts, transmission of TB and progression of latent TB infection to TB in infected contacts should be rare. As noted earlier, other studies report a secondary case rate of between <1% and 4% in contact investigations; as programs progress towards TB elimination, this rate may be further reduced. DNA fingerprinting is most likely to be relevant when a case is suspected of being part of a larger outbreak, for which fingerprints could confirm extensive transmission. This survey suggests that DNA fingerprinting could also be used when an apparent source for an index case is sputum smear-negative, to evaluate whether the smear-negative case could be the true source case, or whether further investigation is needed.
